# Evaluation of paravertebral blocks in improving post-procedural pain and decreasing hospital admission after microwave ablation of liver tumors

**DOI:** 10.1038/s41598-023-36607-1

**Published:** 2023-08-24

**Authors:** Nicholos Joseph, Virginia H. Sun, Avik Som, John Di Capua, Lina Elsamaloty, Junjian Huang, Rafael Vazquez

**Affiliations:** 1 Harvard Medical School, Massachusetts General Hospital, Boston, USA; 2 Division of Vascular and Interventional Radiology, Department of Radiology, Massachusetts General Hospital, 55 Fruit St, Boston, MA 02114 USA; 3 Division of Vascular and Interventional Radiology, Department of Radiology, University of Pennsylvania, Philadelphia, USA; 4 Division of Vascular and Interventional Radiology, Department of Radiology, University of Alabama at Birmingham, Birmingham, USA; 5 Department of Anesthesiology, Massachusetts General Hospital, 55 Fruit St, Boston, MA 02114 USA

**Keywords:** Outcomes research, Quality of life, Pain management

## Abstract

Although ablations are performed with conscious sedation or general anesthesia, microwave ablations can be painful post procedure. Newer analgesic modalities, including regional blocks, have promoted the proliferation of less invasive anesthesia care for ablative procedures. This study evaluates whether bilateral paravertebral blocks reduce the need for additional analgesics in comparison to unilateral blocks in microwave ablations. In this retrospective study, individuals undergoing microwave ablation who underwent unilateral versus bilateral nerve blocks at a single institution from 2017 to 2019 were compared. Categorical variables were analyzed using Pearson’s chi-squared tests. Comparisons of means were completed using multiple T-tests corrected using the Holm-Sidak method with $\alpha$ = 0.05. Regression modeling was used to identify factors related to increased MME (milligram morphine equivalent) usage and post-procedure admission rates. A total of 106 patients undergoing 112 liver MWA procedures were included in this analysis, with patients receiving either a bilateral or unilateral block. Pre-procedural characteristics demonstrated no significant differences in age or gender. Bilateral blocks were associated with decreased usage of gabapentin (14% vs. 0%, p = 0.01) and a lower rate of post-procedure admissions (OR 0.23, p = 0.003). Therefore, when using paravertebral blocks, bilateral blocks are superior to unilateral blocks, as demonstrated by decreased rates of hospital admission and reduced use of systemic neuropathic pain medication. Additionally, reducing post-procedural MME may reduce the rate of admission to the hospital.

## Introduction

Percutaneous thermal ablations have become mainstays in oncologic therapy. However, despite their minimally invasive nature, up to 33% of patients undergoing radiofrequency (RFA) and microwave (MWA) ablations experience significant post-procedural pain as a sequela of post-ablation syndrome^1–5^. Furthermore, intraoperatively, pain management is of the utmost importance. Intraoperative pain can induce physiologic responses, such as shock or bradycardia, which can lead to early termination of the procedure, decrease the intensity of the ablation intraoperatively, and even lead to profound post-operative morbidity^6^. Analgesics, including opioids, are frequently prescribed to alleviate these symptoms, but given concerns of dependence on such agents, other forms of periprocedural pain control have been implemented. Options that have been utilized in non-OR anesthesia (NORA) settings to decrease pain associated with thermal ablations include, but are not limited to, anti-inflammatories, such as NSAIDs and dexamethasone, mixed opioid agonist/antagonists, such as tramadol, and peripheral nerve blocks^7^. In addition to aiding the technical success of a procedure, providing adequate analgesia has demonstrated lower pain scores, increased intraprocedural patient cooperation, decreased readmission rates, and decreased post-procedural pain medication requirements^8,9^.

While the efficacy of these agents in producing a variety of different downstream positive effects is clear, these elements largely have not been evaluated against each other for liver ablations. The present study specifically investigates regional peripheral nerve blocks and whether a unilateral or bilateral paravertebral block is more effective in decreasing post-procedural opioid requirements, utilization of general anesthesia, and decreasing rates of admission to the hospital.

## Materials and methods

This study was approved by the Massachusetts General Brigham Institutional Review Board, completed with a waiver of consent, and HIPAA-compliant. All methods were performed in accordance with the relevant guidelines and regulations, including the Strengthening the Reporting of Observational Studies in Epidemiology (STROBE) checklist for cohort studies. A retrospective study was performed analyzing all patients who underwent liver MWA from January 2017 to December 2019 at a single institution. A total of 112 procedures among 106 patients were performed during this period. Among the procedures performed, 28 underwent ultrasound-guided unilateral thoracic paravertebral blocks (TPVB), and 84 underwent bilateral blocks. A 10-point Visual analogue scale (VAS) score was used to quantify patient pain levels at baseline as well as at post-procedural intervals (initial, 30 min, 60 min, and > 120 min). Data primarily focused on analyzing the reduction of admissions and post-procedural morphine milligram equivalents (MME) with regards to unilateral vs. bilateral blocks. At this institution, the department cycled from no paravertebral blocks to bilateral as an effort to reduce pain. This study explores this transition.

### Management and technique of paravertebral block placement

**Figure 1:**
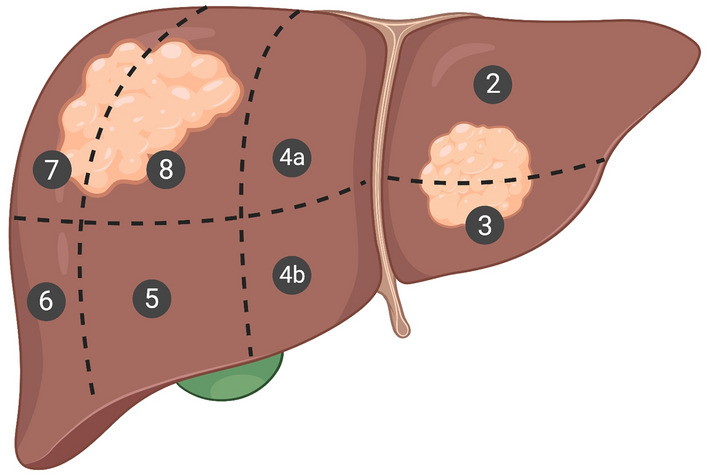
Diagram of liver segments II-VIII. Created with biorender.com.

Selection criteria for performing unilateral versus bilateral blocks was based on anesthesia preference built on lateralization of the tumor. Tumors in liver segment VII generally were considered for unilateral blocks, whereas tumors in segment II, IVa, and most of VIII were given bilateral blocks (see Fig. 1), though over the course of this study patients were more likely to undergo bilateral block placement irrespective of which liver segment was affected. The use of general anesthesia was decided a priori to block administration by the anesthesiologist team based on physician comfort and preference. There was one case in the unilateral block group where the patient was converted to general anesthesia due to pain.

**Figure 2:**
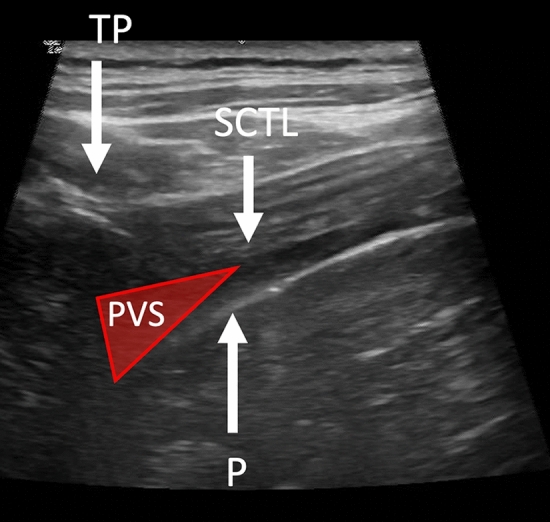
Labeled ultrasound image of paravertebral space (PVS), located between the transverse process (TP), pleura (P), and superior costotransverse ligament (SCTL).

Prior to performing the block placement, the risks, benefits, and alternatives to paravertebral block administration were discussed with each patient, and informed consent was obtained. The patient was placed in prone position and prepared using sterile technique, and the ultrasound machine was positioned appropriately in transverse plane between the T7 and T8 vertebrae. The needle was inserted in-plane from lateral to medial, aiming for the thoracic paravertebral space located at the junction of the pleura and the acoustic shadow underneath the transverse process, as depicted in Fig. 2. Correct placement of the needle was confirmed by the anterior displacement of pleura upon injection of local anesthetic (Supplementary Video 1). Injectate consisted of either bupivacaine, ropivacaine, or mepivacaine (Supplementary Table S1), and was chosen based on the patient’s clinical history, including patient weight and perceived procedure difficulty, as well as preference of the regional anesthesiologist performing the block. Bupivacaine 0.5% or ropivacaine 0.5% were most often chosen based on their potency, while diluted bupivacaine 0.375% was chosen when a better safety profile was desired. Mepivacaine 0.5% was used in situations whereby a rapid onset of analgesia was required.

The same procedure was performed on the opposite side for patients receiving bilateral blocks.

### Pre-operative, intraoperative and post-ablation pain management

Prior to MWA, patients were given a subset of acetaminophen, celecoxib, tramadol, lidocaine, and/or gabapentin, which was decided by the proceduralist based on physician comfort and preference. Intraoperatively, patients received systemic analgesia in the form of dexamethasone, fentanyl, and/or hydromorphone. Following the procedure, patients were offered multi-modal analgesia that generally constituted of acetaminophen, opiates, and ketorolac if renal clearance permitted. Patients received instructions and counseling on use of pain medications prior to discharge. Pre-, intra- and post-operative pain management regimens are summarized in Supplementary Table S1.

### Statistical analysis

A total of 106 patients undergoing 112 liver MWA procedures were included in the analysis. Categorical variables were analyzed with Pearson’s chi-square tests, and continuous variables were performed using unpaired t-tests. A Holm-Sidak correction with α = 0.05 was applied to account for multiple comparisons. Multivariate logistic and linear regressions were used to identify risk factors associated with increased rate of admission and usage of MME, respectively. All statistical tests were done on Prism 7 (GraphPad, San Diego, CA, and Stata), and descriptive statistics were utilized for basic comparison. All analyses were done at the per procedure level. Procedures were stratified according to age, gender, location of tumor, and primary tumor type. VAS was utilized for comparison with respect to pre- and post-procedural pain. Post-operative pain control, use of general anesthesia, and hospital admission rates were compared between patients who underwent unilateral vs. bilateral paravertebral block placement.

### Conference presentation

Material was presented at the 2021 Society for Interventional Radiology Annual Scientific Meeting.

## Results

### Overall outcomes

A total of 112 procedures among 106 patients were analyzed in this study. There were 28 unilateral block placements among 28 unique patients and 84 bilateral block placements among 78 patients. Several individuals underwent more than MWA procedure due to the development of multiple lesions over time. One individual in this study had residual metastatic disease left requiring additional MWA therapy and thus an additional paravertebral block placement. There were no statistically significant differences between age, gender, body mass index, ASA score, mode of anesthesia, pre-procedure labs, primary tumor type, procedure characteristics, pre- and post-procedural pain scoring, intra-operative and post-operative analgesics, and ablation size as measured in Watt-hours between unilateral block and bilateral block groups. Patients in the unilateral group were more likely to receive gabapentin pre-procedure (unilateral vs. bilateral: 14% vs. 0%, p = 0.01) but less likely to receive a lidocaine patch (68% vs. 94%, p = 0.007). As expected, there was an increase in local injectate dosage among patients who received bilateral blocks compared to those who received a unilateral block. For example, among patients who received bupivacaine 0.5%, those in the unilateral block group received on average 24 mL compared to 40.38 mL in the bilateral block group (p = 2.3e−08). Similarly, patients who received ropivacaine 0.5% received 16.25 mL in the unilateral group compared to 37.44 mL in the bilateral group (p = 1.6e−04). Overall patient and procedure characteristics are summarized in Supplementary Table S1.

### Single versus bilateral blocks

Patients who received a bilateral block had a lower admission rate compared to those with only a unilateral block (unilateral vs. bilateral: 50% vs. 20%). This was a statistically significant difference (p = 0.003) as determined by multiple logistic regression (Table 1). Among the 14 individuals who required admission following unilateral block placement, seven had pain control listed as reason for admission, while the remaining individuals were admitted for observation. Of the 17 individuals requiring admission in the bilateral block group, four had pain control listed as a reason for admission. Additional reasons for admission in the bilateral block group included observation (7/17), hematoma formation secondary to MWA (2/17), flash pulmonary edema (1/17), non-pain related discomfort (1/17), post-operative delirium (1/17), and work-up of other medical conditions (1/17).

**Table 1:** Multiple logistic regression (odds ratio) for prediction of admission.

	Coefficient (SE)	Odds ratio (SE)
Age	0.025 (0.025)	1.025 (0.025)
General anesthesia	− 1.569 (1.533)	0.208 (0.319)
MAC	− 1.635 (1.476)	0.195 (0.288)
Male	− 0.277 (0.479)	0.758 (0.363)
Hydrodissection	− 0.319 (0.502)	0.727 (0.365)
Bilateral block (relative to unilateral)	− 1.469*** (0.494)	0.230*** (0.114)
Constant	0.242 (2.306)	1.274 (2.938)

*SE* standard error.

***p-value = 0.003.

Those patients who underwent bilateral nerve blocks fared the best when it came to decreased utilization of post-procedural morphine equivalents, with an average utilization of 3.70 ± 7.44 MME. By comparison, those who underwent unilateral nerve block had an average MME usage of 5.56 ± 8.22 units, raising concern that those patients who underwent unilateral nerve blocks exhibited a greater need for pain control. This difference was not significant as determined by multiple linear regression (Table 2, p = 0.08). When comparing the amount of post-procedural MME required, those who required admission necessitated a higher average need for MME (22.10 units) versus those who received post-procedural MME but did not require admission (11.97 units).

**Table 2:** Multiple linear regression of morphine equivalent vs various variables.

Variables	MME (SE)
Age	− 0.005 (0.078)
General anesthesia	− 0.836 (5.852)
MAC	− 1.980 (5.657)
Male	− 0.300 (1.582)
Hydrodissection	− 1.610 (1.668)
Bilateral block (relative to unilateral)	− 1.752 (1.800)
Constant	8.172 (8.199)

*SE* standard error.

**Figure 3:**
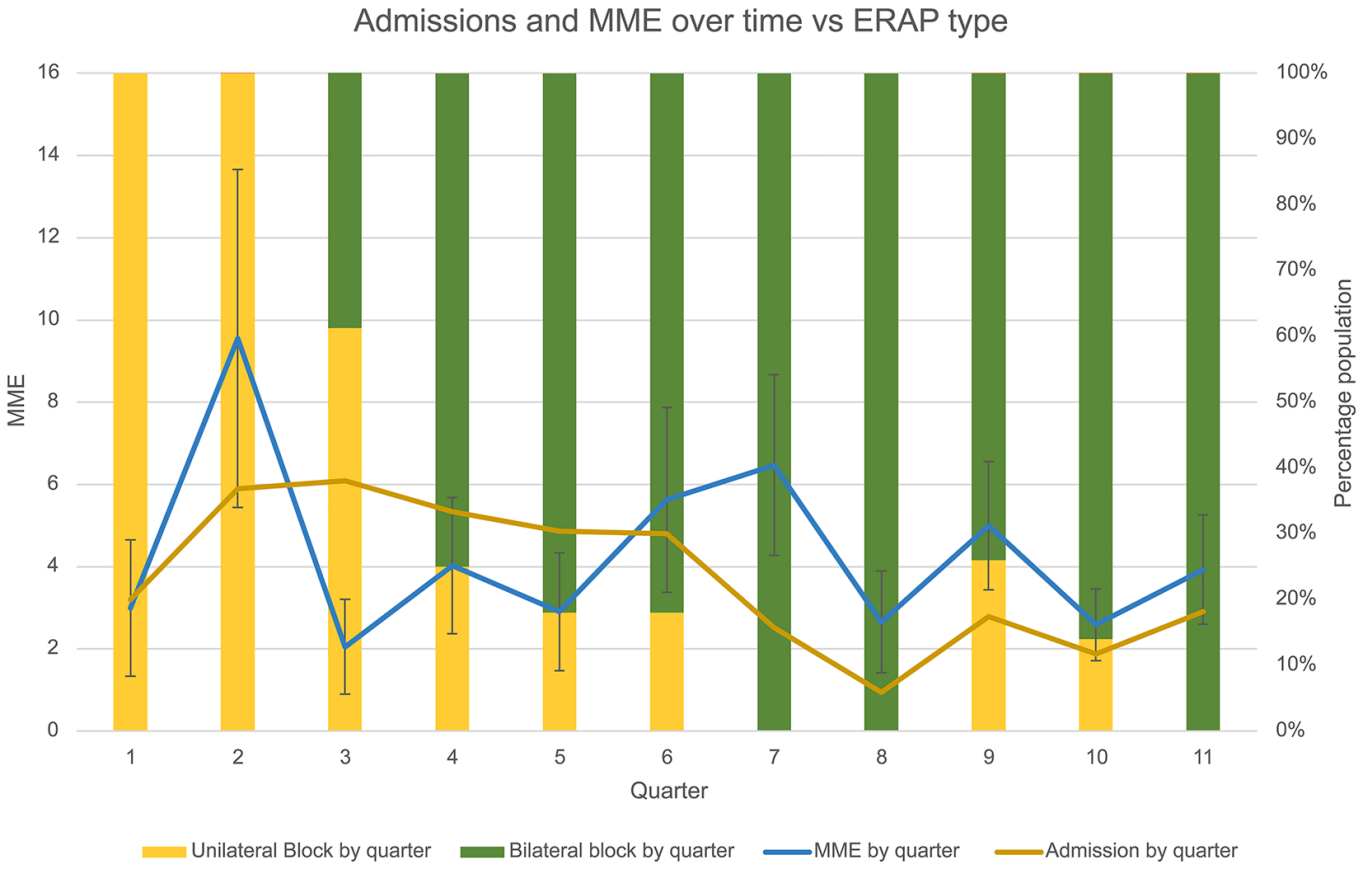
Change in admissions with the gradual shift in block type by quarter.

Finally, there was a statistically significant decrease in the proportion of unilateral blocks performed over time with a concomitant rise in the proportion of bilateral blocks (Fig. 3, R^2^ = 0.6743, p = 0.023), reflecting the institution’s practice in switching from placing unilateral paravertebral blocks to bilateral over the course of the study period.

## Discussion

This single-institution retrospective analysis investigated the effect of paravertebral blocks on rate of admission and total opioid administration. The results of this study revealed that utilization of bilateral paravertebral blocks significantly reduced the rate of hospital admissions (50% vs. 20%, p = 0.003), including admissions for post-procedural pain (25% vs. 5%, p = 0.002), compared to unilateral paravertebral blocks. Furthermore, there was a clinically significant lower burden of opioids required in patients who underwent bilateral nerve blocks as demonstrated by the decreased MME utilization (5.56 vs. 3.70, p = 0.08). The lack of statistical significance is likely due to lack of power, as well as a relatively wide standard deviation in starting opioid needs, which may reduce the apparent efficacy of total MME even if pain itself is improved.

In this study, patients in the unilateral and bilateral block groups also received multimodal analgesia pre-procedure, intra-operatively, and post-operatively. There was a statistically significant difference in pre-procedure gabapentin and lidocaine patch usage across the two groups. Patients in the bilateral block group were more likely to use lidocaine patch (14% vs. 0%, p = 0.01) and less likely to require gabapentin (68% vs. 94%, p = 0.007). This finding suggests that patients who receive bilateral paravertebral blocks rather than unilateral may require fewer systemic agents to treat neuropathic pain in favor of local agents, thereby reducing likelihood of developing adverse drug reactions^10^.

The utility of paravertebral blocks in reducing patient pain is well documented in the literature. Thoracic paravertebral blocks (TPVB), for instance, have been shown to be well tolerated and provide symptomatic relief of pain^11^. Microwave ablation of hepatic lesions following these blocks resulted in minimal pain, and post-procedurally, patients reported minimal pain within the first 24 h and required few analgesics.

A considerable amount of literature continues to reiterate the benefits and superiority of TPVB for percutaneous radiofrequency tumor ablation (PRFA)^11–13^. However, although numerous studies have discussed the benefits of TPVB, consideration of utilization of general anesthesia often arises and may be appropriate in certain scenarios. A general consideration of the strengths and weaknesses of general anesthesia (GA) in the setting of PRFA of hepatocellular carcinoma (HCC) has been previously reported^14^. In their study, advantages included an overall decreased number of sessions to accomplish complete tumor ablation, as well as decreased hospitalization, reduced systolic pressure, and reduced hepatic blood flow, thus increasing tumor ablation diameter. Disadvantages of GA included increased risk of myocardial infarction, malignant hyperthermia, stroke, the limitations of requiring necessary equipment, and pre-procedural risk stratification^14^. In the appropriate clinical scenario, these and other considerations should be made.

This study demonstrated that bilateral paravertebral blocks were preferred over unilateral. While the precise innervation of the hepatic parenchyma is not well understood, perception of pain is thought to be mediated by a complex network of both visceral and somatic afferent fibers, with postoperative pain usually having a dominant somatic component^15^. Afferent fibers arising from the liver parenchyma have demonstrated partial crossing to the contralateral side at the thoracic level, which may explain why bilateral blocks provide better anesthesia coverage^16^. Major limitations in unilateral blocks have been described, including “failure to achieve total visceral anesthesia” due to lack of proper involvement of both the parasympathetic and contralateral sympathetic nerve fibers^17^. Thus, despite their less invasive and time-saving elements, unilateral blocks fall short in their ability to adequately treat and prevent patient pain. However, the relatively more invasive aspect cannot be overlooked: a balance must be struck in this regard, and the clinician must use their gestalt to determine the most appropriate option in each individual case. This fine balance of achieving proper sedation and analgesia while maintaining appropriate blood pressures and minimizing patient pain is, of course, a critical topic in interventional radiology that continues to be explored^18–20^.

Limitations of this study include both its retrospective nature and a limited number of cases overall. Of the 112 procedures analyzed in this study, only 28 were unilateral block placements. This discrepancy between the number of recipients who received a bilateral block compared to those that received a unilateral block reduces the power of the study. Therefore, findings that were not statistically significant, such as the decreased MME among patients receiving bilateral blocks, may still be clinically significant. However, although the results of the study favor the use of bilateral paravertebral blocks, each case must be considered individually, and the interventionist must adapt to a variety of situations in which local anesthesia may be preferred over regional or general anesthesia. Further inquiry with a larger sample size in a prospective study design is necessary to elucidate and bolster the findings in this report.

## Conclusion

By the merits of this study, bilateral paravertebral blocks were superior to unilateral paravertebral blocks in reducing rates of hospital admission for liver microwave ablation and usage of systemic agents to treat neuropathic pain. Of note, reducing post-procedural MME reduced the admission rate for pain.

### Supplementary Information

Supplementary Table 1.

Supplementary Video 1.

## Data Availability

Raw data were generated at Massachusetts General Hospital. Derived data supporting the findings of this study are available from the corresponding author (RV) on request.
