# Supplementary material for: Evaluation of paravertebral blocks in improving post-procedural pain and decreasing hospital admission after microwave ablation of liver tumors
Source: Sci Rep. 2023 Aug 24;13:13854. doi: 10.1038/s41598-023-36607-1 (PMC10449898; doi:10.1038/s41598-023-36607-1)
Supplement: Supplementary file 1 — Supplementary Table 1. [file 41598_2023_36607_MOESM1_ESM.docx]

**Evaluation of Paravertebral Blocks in Improving Post-procedural Pain and Decreasing Hospital Admission After Microwave Ablation of Liver Tumors.**

**Nicholos Joseph, BA**^1*^, **Virginia H. Sun, BS**^1*^**, Avik Som, MD, PhD**^2*^**, John Di Capua, MD**^2^**, Lina Elsamaloty, MD**^3^**, Junjian Huang, MD**^4^**, Rafael Vazquez, MD**^5^

^1^Harvard Medical School

Massachusetts General Hospital

^2^Department of Radiology, Division of Vascular and Interventional Radiology

Massachusetts General Hospital

^3^Department of Radiology, Division of Vascular and Interventional Radiology

University of Pennsylvania

^4^Department of Radiology, Division of Vascular and Interventional Radiology

University of Alabama at Birmingham

^5^Department of Anesthesia

Massachusetts General Hospital

* These authors contributed equally: Nicholos Joseph, Virginia H. Sun and Avik Som.

**Supplementary Table 1. Procedure Characteristics,** * p<.05 using chi-squared and multiple t-test with Holm-Sidak Correction. All values are based per procedure, unless otherwise noted.

|  | **Unilateral Block + MM**  **N = 28 patients**  **N = 28 procedures** | | | **Bilateral Block + MM**  **N = 78 patients**  **N = 84 procedures** | | |
| --- | --- | --- | --- | --- | --- | --- |
|  | Mean | SD | N | Mean | SD | N |
| **Unique Patients** |  |  | 28 |  |  | 78 |
| **Unique Procedures** |  |  | 28 |  |  | 84 |
| **Age** | 67.82 | 10.43 | 28 | 67.08 | 9.90 | 84 |
| **Male** | 75% |  | 28 | 56% |  | 84 |
| **Female** | 25% |  | 28 | 44% |  | 84 |
| **BMI** | 26.39 | 4.91 | 28 | 29.06 | 5.64 | 83 |
| **ASA Score** | 2.76 | 0.44 | 21 | 2.82 | 0.39 | 82 |
| **Ablation Size (Watt-hours)** | 14.48 | 9.39 | 27 | 13.66 | 12.39 | 81 |
| **Mode of Anesthesia** |  |  |  |  |  |  |
| GETA | 29% |  | 28 | 15% |  | 84 |
| LMA | 4% |  | 28 | 2% |  | 84 |
| MAC | 68% |  | 28 | 80% |  | 84 |
| **Pre-Procedure Labs** |  |  |  |  |  |  |
| INR | 1.11 | 0.10 | 28 | 1.11 | 0.10 | 84 |
| Prothrombin Time | 14.25 | 1.03 | 28 | 14.12 | 1.09 | 84 |
| Platelets | 142.93 | 57.22 | 28 | 161.30 | 80.14 | 84 |
| Creatinine | 0.95 | 0.27 | 27 | 0.97 | 0.28 | 83 |
| **Premedication** |  |  |  |  |  |  |
| Acetaminophen | 86% |  | 28 | 94% |  | 84 |
| Celecoxib | 54% |  | 28 | 56% |  | 84 |
| Tramadol | 43% |  | 28 | 58% |  | 84 |
| Lidocaine patch* | 68% |  | 28 | 94% |  | 84 |
| Gabapentin* | 14% |  | 28 | 0% |  | 84 |
| **Local Injectate** |  |  |  |  |  |  |
| BUPI 0.375% only | 20 | 0 | 2 | N/A | N/A | 0 |
| BUPI 0.5% only* | 24 | 6.76 | 20 | 40.38 | 7.98 | 32 |
| ROPI 0.5% only* | 16.25 | 4.79 | 4 | 37.44 | 7.97 | 43 |
| MEPI 1.5% only | N/A | N/A | 0 | 40 | N/A | 1 |
| BUPI 0.5% and MEPI 1.5% | 10, 10 | N/A | 1 | 21.25,17.5 | 2.5, 5 | 4 |
| **Primary Tumor** |  |  |  |  |  |  |
| Hepatocellular Carcinoma | 71% |  | 28 | 67% |  | 84 |
| Cholangiocarcinoma | 0% |  | 28 | 6% |  | 84 |
| Colon | 0% |  | 28 | 2% |  | 84 |
| Melanoma | 0% |  | 28 | 2% |  | 84 |
| Other Cancer | 29% |  | 28 | 23% |  | 84 |
| **Intra-Operative Characteristics** |  |  |  |  |  |  |
| # Burns | 2.10 | 1.02 | 20 | 1.96 | 1.04 | 78 |
| Hydro-dissection | 29% |  | 28 | 31% |  | 84 |
| Hydro-dissection volume (mL) | 327.92 | 702.76 | 24 | 124.79 | 339.24 | 71 |
| Local dose (mL) | 22.50 | 6.78 | 26 | 38.23 | 9.03 | 77 |
| Dexamethasone | 6.32 | 5.06 | 25 | 5.99 | 4.55 | 84 |
| Intraoperative fentanyl (mcg) | 142.13 | 64.59 | 27 | 115.06 | 66.48 | 82 |
| Intraoperative hydromorphone (mg) | 0.24 | 0.56 | 27 | 0.08 | 0.23 | 79 |
| **Pain Scoring** |  |  |  |  |  |  |
| Pre-procedure VAS | 0.29 | 1.08 | 24 | 0.72 | 1.93 | 77 |
| VAS PACU arrival | 1.80 | 2.87 | 25 | 1.45 | 2.69 | 73 |
| VAS 30 min | 2.09 | 3.09 | 23 | 1.58 | 3.02 | 66 |
| VAS 60min | 2.14 | 2.73 | 21 | 2.42 | 3.38 | 73 |
| VAS>120 min | 2.22 | 2.47 | 23 | 2.30 | 3.01 | 76 |
| **Post-Operative Events** |  |  |  |  |  |  |
| Tramadol (mg) | 1.85 | 9.62 | 27 | 7.83 | 22.74 | 82 |
| Hydromorphone (mg) | 0.44 | 1.47 | 27 | 0.32 | 1.31 | 82 |
| Oxycodone (mg) | 2.59 | 4.68 | 27 | 1.19 | 2.97 | 83 |
| MME (mg) | 5.56 | 8.22 | 27 | 3.70 | 7.44 | 80 |
| Post procedure ketorolac (mg) | 6.67 | 10.47 | 27 | 8.49 | 11.76 | 82 |
| Admission | 50% |  | 28 | 20% |  | 83 |
|  |  |  |  |  |  |  |

BMI – Body Mass Index

ASA – American Society of Anesthesiology score

GETA – general endotracheal anesthesia

LMA – laryngeal mask airway

MAC – monitored anesthesia care

BUPI – bupivacaine

ROPI – ropivacaine

MEPI – mepivacaine

VAS – visual analog scale

PACU – post-anesthesia care unit

MME – milligrams morphine equivalents
